# Broadband Impedance Matching for Immersed CMUTs: An End-to-End Design-to-Measurement Validation Framework

**DOI:** 10.3390/s26051546

**Published:** 2026-03-01

**Authors:** Gabriel Guerreiro, Martin Angerer, Edmond Cretu

**Affiliations:** Department of Electrical and Computer Engineering, The University of British Columbia, Vancouver, BC V6T 1Z4, Canada; m.angerer@ubc.ca (M.A.); edmondc@ubc.ca (E.C.)

**Keywords:** front-end electronics, CMUT, Bode–Fano criterion, Chebyshev, polyCMUT, broadband impedance matching

## Abstract

Capacitive micromachined ultrasonic transducers (CMUTs) are a promising alternative to conventional piezoelectric transducers, offering superior design flexibility and broadband operational characteristics. However, their clinical and practical deployment is constrained by elevated driving voltages and limited acoustic power output, particularly when producing CMUTs based on polymers. This paper presents an end-to-end, measurement-driven experimental validation strategy for designing passive broadband impedance-matching networks that enhance transmitted acoustic power in immersed CMUT arrays while preserving bandwidth. Matching topologies are synthesized to operate near the theoretical Bode–Fano limit, and robustness to component tolerances is quantified through Monte Carlo yield analysis using realistic off-the-shelf component variations. The matching networks are then implemented and experimentally validated under representative unipolar pulse excitation, with far-field acoustic pressure characterized in both time and frequency domains and compared against numerical predictions. The results show that the optimized impedance matching increases transmit power by a factor of 2.1 at the cost of a 40% fractional-bandwidth reduction. These findings establish a directly applicable, validated framework for broadband impedance matching in polymer CMUT arrays and support its use as a cost-effective approach for ultrasound imaging and therapeutic systems.

## 1. Introduction

Capacitive micromachined ultrasonic transducers (CMUTs) have matured in recent years and now rival piezoelectric devices as a leading ultrasound transducer technology, driven by their superior acoustic performance [1], design flexibility [2] and broadband frequency response [3]. CMUTs are essentially microscopic drums fabricated by micromachining silicon layers and actuated via electrostatic forces. One drawback, compared to traditional piezoelectric transducers, is their relatively limited output pressure due to the limited membrane volume and effective deflection [4]. Achieving the MPa-level pressures required for applications such as medical imaging typically necessitates high bias and driving voltages [5,6]. This limits their use in low-power or battery-operated systems such as wearables [7]. Polymer-based CMUTs (polyCMUTs) offer additional advantages in rapid prototyping and cost, yet they face further output-power limitations due to the lower dielectric strength of their insulating layers.

Several approaches have been proposed to increase CMUT output pressure, including geometric optimization, square-wave excitation, high-permittivity insulating layers, the use of center masses, and electrical impedance matching, as recently reviewed in 2025 [8]. Among these, front-end impedance matching is particularly effective for maximizing transmitted acoustic power because it can improve power transfer from the electrical source to the immersed CMUT load while introducing limited bandwidth reduction to preserve broadband operation. This approach has the advantage that the transducer design itself does not need to be modified, avoiding complex design iterations and months-long microfabrication cycles. However, it requires placing passive electrical components (e.g., inductors) in close proximity to the transducer.

For CMUTs, various passive matching network topologies have been explored for this purpose, ranging from high-Q single-inductor matching [9,10,11] to low-Q LC-ladder networks [12] composed entirely of passive components, while more elaborate approaches adopted higher-order Butterworth filters to broaden the bandwidth [13]. Alternative active approaches employ negative capacitance circuits to effectively cancel the intrinsic capacitance $C_{0}$ [14] of the CMUT, though these tend to increase circuit power consumption and noise due to the need of active components. Overall, the complexity of a potential impedance matching network is determined by the application of the ultrasound system.

In high-intensity focused ultrasound (HIFU) therapy, for example, the primary objective is to deliver sustained high-power continuous waves to achieve effective thermal ablation [15,16]. In this context, higher output power is generally prioritized over a broad bandwidth. Hence, a single inductor matching would be enough to achieve the system’s requirements in such cases. However, broadband impedance-matching networks can be advantageous for achieving the required pressure levels over a range of drive frequencies [17].

For other applications such as tissue harmonic imaging [18] and medical imaging combined with HIFU therapy [19], a broader bandwidth is beneficial to achieve better image quality and more localized focal points. Another interesting example is ultrasound-based communication systems for medical implants, where impedance matching can enable high acoustic output pressure (for deep acoustic penetration) while maintaining a low bit-error rate through better SNR and limited pulse ringing [20,21,22].

The examples above highlight the need for a practical, measurement-driven tool that allows designers to trade bandwidth for transmitted power without modifying the CMUT structure. This paper presents an end-to-end experimental validation of broadband electrical impedance matching for immersed CMUT arrays, combining robustness assessment and acoustic performance verification under pulse excitation. Starting from the measured input impedance of an in-house polyCMUT array, we extract an equivalent small-signal model, synthesize matching networks targeting operation close to the theoretical Bode–Fano criterion [23], and quantify robustness using Monte Carlo yield analysis with realistic component tolerances. We then implement and compare narrowband and broadband networks and validate their performance using representative unipolar pulses, reporting far-field acoustic pressure in both the time and frequency domains. The proposed approach is designed to sacrifice only the minimum bandwidth required to achieve a meaningful increase in transmitted power.

The remainder of this paper is organized as follows. Section 2 presents the proposed broadband impedance-matching methodology and its application to the polyCMUT device. Section 3 reports the experimental results for three matching scenarios. Section 4 discusses the main findings, and Section 5 concludes the paper.

## 2. Materials and Methods

Perfect conjugate matching to a complex load yields maximum power transfer only at the frequency where the source impedance equals the complex conjugate of the load impedance. In practice, this corresponds to a high-*Q* condition: power transfer is maximized near resonance but degrades rapidly away from it. When a wider operating band is required, the matching network can be designed with a lower effective *Q*, trading peak transferred power for bandwidth. In that case, the match is no longer exact and a portion of the incident power is reflected back to the source.

The appropriate bandwidth–power compromise is application-dependent. Many prior approaches impose minimum bandwidth and power targets, but do not explicitly assess whether the achieved solution is optimal—i.e., whether it delivers the maximum possible transferred power over a prescribed bandwidth, or conversely, the maximum attainable bandwidth for a given power-transfer level. The Bode–Fano criterion provides a theoretical boundary on this trade-off by linking the achievable reflection performance to the causality constraints of passive matching networks.

The Bode–Fano limit is derived under three key assumptions: passivity, linearity, and time invariance [24]. Accordingly, we design the matching network using only passive elements (inductors and capacitors) between the source and the load. Around the small-signal operating point, the CMUT input impedance is approximated by an equivalent RC load, and the system can be treated as linear and time-invariant within the small-signal regime.

### 2.1. Impedance Matching Network Design

The general procedure to estimate the CMUT parameters and design the impedance matching circuit is based on [25], where a Chebyshev filter is synthesized to match the source and load impedances. The aim is to match a usual 50 ohms output impedance from signal generators to the CMUT impedance in IPA. Here are the general steps:

1. Measure the immersed CMUT parameters.
2. Define the filter parameters.
3. Select the filter type.
4. Select the filter order.
5. Tune the filter transfer function to satisfy the Bode–Fano criterion.
6. Compute the roots of the reflection coefficient.
7. Synthesize the matching network.

### 2.2. CMUT Device and Electrical Characterization

To demonstrate the effectiveness of the proposed broadband impedance-matching approach, we synthesized the corresponding matching networks using the procedure described above and implemented them for a polyCMUT array.

#### 2.2.1. Device and Microfabrication

The polyCMUT linear array used to evaluate the different impedance matching networks were fabricated in-house in our Advanced Materials Process Engineering Laboratory at UBC using a sacrificial release process [26]. The array comprises 16 elements, each containing 540 circular cells or drums. The membrane and top-electrode radii are 48 μm and 40.8 μm, respectively.

Producing the transducer chips requires three main microfabrication steps: First, photo-lithographic patterning of a photosensitive polymer. We use SU-8 (Kayaku Advanced Materials, Westborough, MA, USA) due to its excellent properties in producing mechanically stable microscopic structures with high aspect ratios [27]. Next, thin-film metal deposition using physical vapor deposition, followed by patterning and liftoff. Finally, the microscopic drums are released via critical point drying after etching a thin sacrificial LOR-1A layer (Kayaku Advanced Materials, Westborough, MA, USA), using low concentrations of AZ 300 MIF (Merck Performance Materials GmbH, Wiesbaden, Germany). Producing the required functional layers is entirely additive, meaning that no silicon-based materials require etching. This eliminates the use of highly concentrated or toxic chemicals, and enables rapid fabrication cycles (<1 day). As substrate, we used a 4-inch, 0.5 mm thick silicon wafer with 500 nm thermal oxide.

**Figure 1:**
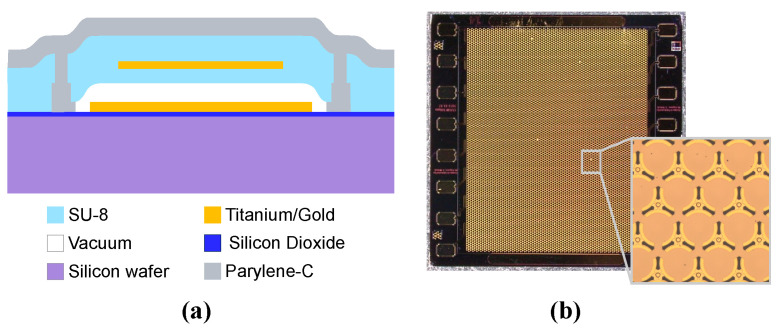
PolyCMUTs used in this study: (**a**) schematic cross section of a single cell with functional layers, (**b**) photograph of 16 channel linear array with magnification of several cells.

After the sacrificial release, the polyCMUT chips were glued onto custom Printed Circuit Boards (PCBs), and electrically connected with wire bonds. To seal the etched cavities, a conformal Parylene-C coating (SCS Labcoater 2, Specialty Coating Systems, Indianapolis, IN, USA) was foreseen. Figure 1 gives a schematic cross section of a single cell after sealing the etched cavity, and an overview picture of the 16 channel linear array chip used in this study, with partial magnification. The final layer thicknesses are listed in Table 1. More detailed information on the overall design process for polyCMUTs is provided in [28], while comprehensive details on the applied microfabrication processes, materials, and equipment can be found in [29].

#### 2.2.2. Step 1: Measure the Immersed CMUT Parameters

The required parameters can be extracted using an impedance analyzer or a vector network analyzer (VNA). These measured values are then used to populate an equivalent-circuit model, enabling subsequent simulations of device performance. In this work, the device is characterized and operated in its linear regime.

**Figure 2:**
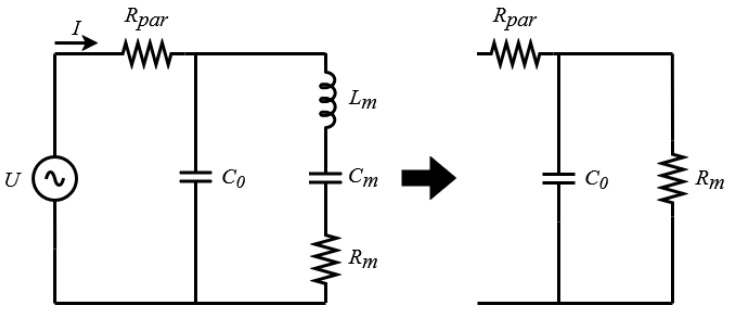
CMUT BVD equivalent circuit on the **left**, and its simplified version in immersion at resonance frequency on the **right**.

The CMUT under small-signal excitation is typically modeled using the Mason equivalent circuit, a linear formulation that couples the electrical, mechanical, and acoustic domains through ideal transformers [30]. In this work, we adopt the Butterworth–Van Dyke (BVD) representation shown in Figure 2 because it enables direct parameter extraction from impedance/admittance measurements and provides a compact model suitable for impedance-matching synthesis [31,32,33]. Since the BVD elements are identified from the measured input impedance in the intended operating condition, deviations from the ideal thin, perfectly clamped plate behavior, such as thick-plate effects and boundary compliance previously analyzed for polymer CMUTs in [23], are implicitly embedded in the fitted lumped parameters. The BVD model comprises the static capacitance $C_{0}$, a motional branch ($L_{m}$, $C_{m}$, $R_{m}$), and a series parasitic resistance $R_{par}$. For immersed operation near the fundamental resonance, radiation loading and viscous losses yield a low-*Q* response that is captured by the motional branch.

Moreover, the simplified version in Figure 2 assumes that the acoustic loading around the operating frequency is captured through an equivalent lumped impedance. This simplification is appropriate over a wide but limited band in which the radiation impedance can be approximated to a single-mode radiator. For increased broader bandwidths, however, the radiation impedance becomes more frequency-dependent, and additional effects, such as dispersion, inter-element coupling, and higher-order membrane modes, can alter the effective input impedance seen by the matching network [34]. As a result, the achieved reflection coefficient and bandwidth may deviate from the predictions of a lumped, frequency-invariant loading model [35]. A more detailed assessment of how these effects influence the matching network is left for subsequent investigation.

**Figure 3:**
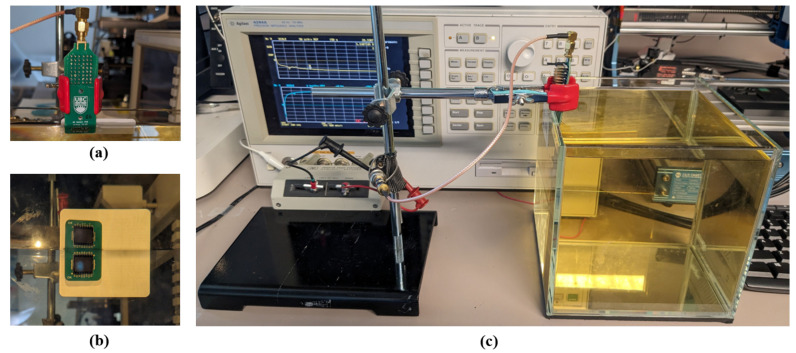
Instrumentation setup for immersed measurements: (**a**) Back side of the PCB before immersion in IPA, (**b**) polyCMUT array immersed and facing the liquid medium, and (**c**) the complete measurement assembly. The tall IPA column ensures operation under far-field conditions.

**Figure 4:**
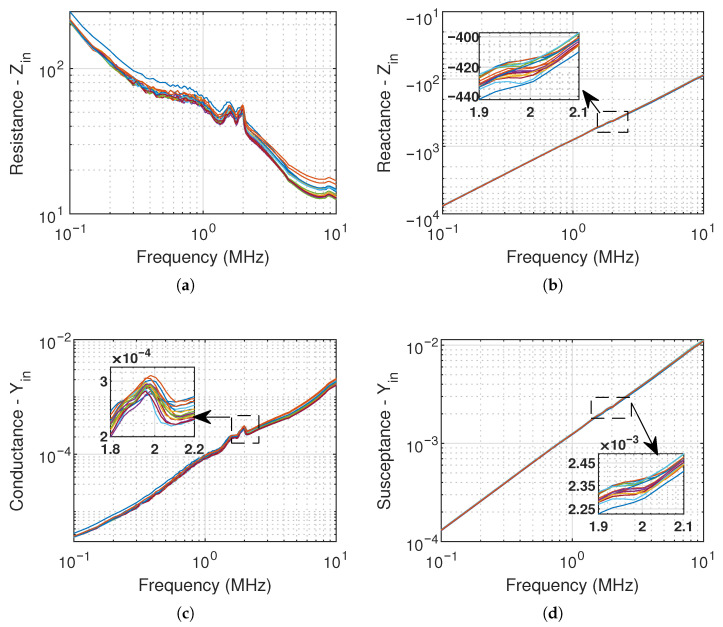
Measured electrical input impedance of the 16 CMUT’s elements. (**a**) Resistance, (**b**) reactance. (**c**) conductance, and (**d**) susceptance.

The electrical impedance $Z_{in}$ and admittance $Y_{in}$ of the 16 elements were measured in immersion using an impedance analyzer (Agilent 4294A, Keysight Inc., Santa Rosa, CA, USA). The frequency sweep spans 100 kHz to 10 MHz. We applied a 40 V DC voltage to bias the polyCMUTs. The measurement setup is depicted in Figure 3. The real and imaginary parts of $Z_{in}$ and $Y_{in}$ are shown in Figure 4. We applied open-short-load compensation before the measurement to compensate for parasitics (e.g., capacitance of the setup).

To extract the component values of the equivalent circuit shown in Figure 2 under immersed conditions, the mean characteristics across the 16 array elements are used. Using the mean significantly simplifies the network by allowing the same passive electrical components to be used across all channels. This simplification can be justified by a low inter-element variability reported in Table 2 for five key characteristics, where the standard deviation of $f_{r}$, $C_{0}$, $G_{p}$, and $B_{p}$ is below 4%, and of $R_{par}$ is below 9%.

The parasitic series resistance, $R_{\mathrm{par}}$, is typically estimated at frequencies well above the operating range. In this work, it is extracted from Re{Zin} at 10MHz. Similarly, the static capacitance, $C_{0}$, is obtained from Im{Zin} at 10MHz.

The motional capacitance, $C_{m}=26.46\;\mathrm{pF}$, is obtained from the difference between the mean capacitance values extracted from Im{Zin} at 100kHz and 10MHz. The motional inductance, $L_{m}=243.94\;\mu H$, is then determined using the mean resonance frequency $f_{r}=1.98\;\mathrm{MHz}$. The resonance frequency $f_{r}$ is identified at the peak of the conductance, since the peak of Re{Zin} occurs closer to the anti-resonance frequency.

For operation in a medium with relatively high acoustic impedance, the plate radiation loading yields a low-*Q* response; near the fundamental resonance, this behavior can be approximated by a parallel RC model [13], as illustrated on the right of Figure 2.

To estimate $R_{m}$ in immersion, we use the admittance representation and extract the parallel conductance and susceptance at $f_{r}$: $G_{p}=287.43\;\mu S$ and $B_{p}=2.33\;\mathrm{mS}$. These quantities are obtained from the measured admittance and therefore already include the effect of $R_{\mathrm{par}}$.

The equivalent series resistance $R_{m}$ accounts for motional losses at resonance. It reduces to $R_{m}=1/G_{p}$ only when $R_{\mathrm{par}}=0\;\Omega$. For nonzero $R_{\mathrm{par}}$, $R_{m}$ is computed as follows:

(1) $$G_{pe}\;=\;ℜ\left\{\frac{G_{p}+iB_{p}}{\;1-R_{par}\;\left(G_{p}+iB_{p}\right)}\right\}$$

The effective motional resistance is computed as $R_{m}=1/G_{pe}={\left(212.76\;\mu S\right)}^{-1}=4.70\;k\Omega$, which is higher than the uncorrected value $1/G_{p}={\left(287.43\;\mu S\right)}^{-1}=3.48\;k\Omega$. Using $G_{pe}$ therefore yields an effective $R_{m}$ that is consistent with the simplified equivalent circuit in Figure 2, which explicitly includes the series parasitic resistance $R_{\mathrm{par}}$. The influence of $R_{\mathrm{par}}$ on $B_{p}$ is negligible in the present operating range and is neglected.

**Figure 5:**
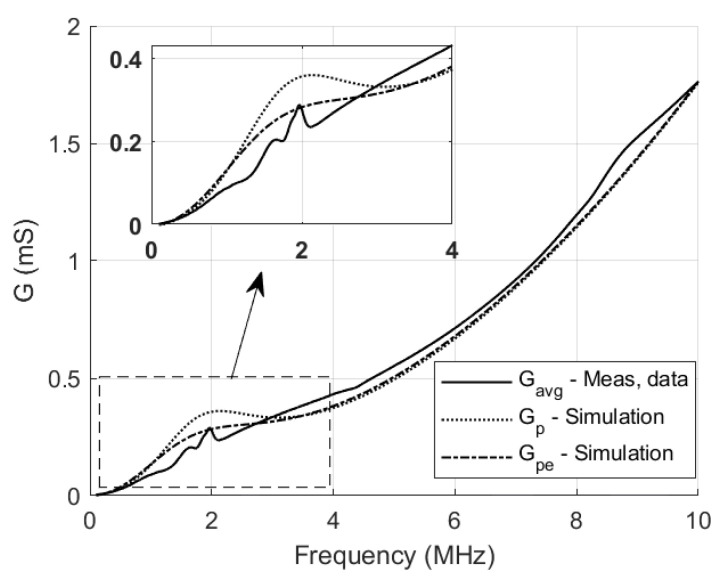
Measured versus simulated data of conductance. It can be noted that the conductance curve after the correction shows a stronger agreement around the resonance frequency.

Figure 5 compares the measured conductance $G_{p}$ with the conductance predicted by the equivalent-circuit model before and after the $G_{p}$ correction, evaluated at the resonance frequency $f_{r}=1.98\;\mathrm{MHz}$.

#### 2.2.3. Step 2: Define the Filter Parameters

The filter center frequency is set to 1.98 MHz, corresponding to the measured immersed resonance of the CMUT used in our experiments. The fractional bandwidth FBW is selected based on the maximum allowable ripple and the filter order, which in turn depends on the degree of mismatch between the source and load impedances. In this work, a FBW of 50% is adopted as a practical reference point for the design.

#### 2.2.4. Step 3: Select the Filter Type

To design the impedance-matching network, we selected a Chebyshev type I response, which exhibits equiripple behavior in the passband. This choice reflects a practical compromise among transition sharpness, circuit complexity, and sensitivity to component tolerances [36]. Other responses (e.g., Butterworth or elliptic) could also be used; however, a detailed comparison of alternative filter topologies is beyond the scope of this paper.

Because the goal of this work is to provide a versatile design tool for application-specific requirements, we adopt the concept of normalization to transform a low-pass prototype filter into a bandpass filter [37].

The prototype element values are first normalized to a unity cutoff frequency 1 rad/s and a 1Ω load resistance, then it is transformed into a bandpass configuration, and finally scaled to the desired frequency and impedance specifications.

We start with the transfer function for the Chebyshev type I low-pass prototype as given by Equation (2).

(2) $$|S_{21}^{\prime }{|}^{2}=\frac{1}{1+\delta +\epsilon^{2}C_{n}^{2}\left(\omega^{\prime }\right)}$$

(3) $$C_{n}=\left\{\begin{array}{cc} cos(n^{\prime }cos^{-1}\left(\omega^{\prime }\right)) & \mathrm{for}\;|\omega^{\prime }|\leq 1\\ cosh(n^{\prime }cosh^{-1}\left(\omega^{\prime }\right)) & \mathrm{for}\;|\omega^{\prime }|\geq 1 \end{array}\right.$$

where $|S_{21}^{\prime }{|}^{2}$ is the magnitude of the forward transmission coefficient, δ is included to avoid singularities of Equation (7) on the real-frequency axis [25], $\epsilon^{2}$ controls the amplitude of the ripple, $C_{n}$ is the characteristic function for the Chebyshev polynomial (3), $\omega^{\prime }$ is the sinusoidal frequency variable scaled such that the cutoff frequency equals unity, and $n^{\prime }$ is the number of reactive elements in the network.

The relationship among the parameters $\epsilon^{2}$, δ, and termination ratio $\left(R_{m}/R_{s}\right)$ is obtained from Equation (2) by imposing the DC condition (ω=0), under which the power-loss ratio is given by Equation (4).

(4) $$\frac{1}{|S_{21}{\left(0\right)|}^{2}}=\frac{R_{m}+R_{s}}{4R_{m}R_{s}}=1+\delta +\epsilon^{2}C_{n}\left(\omega^{\prime }\left(0\right)\right)$$

The next step is to transform the lowpass prototype into a bandpass filter by using the change of frequency variable [25]. We start by calculating the lower and upper cutoff frequencies of the transformed filter. For a FBW of 50% centered at 1.98 MHz, the frequency band goes from 1.845 MHz to 2.475 MHz. Then, we proceed with the normalized lower and upper cutoff frequencies $\omega_{a}$ and $\omega_{b}$, respectively:

$$\begin{array}{ccc} \; & \omega_{a}=1-\frac{FBW}{2}\; & \omega_{b}=1+\frac{FBW}{2} \end{array}$$

From $\omega_{a}$ and $\omega_{b}$, we compute the normalized center frequency $\omega_{0}$ and the auxiliary parameter *A*.

$$\begin{array}{ccc} \; & \omega_{0}=\sqrt{\frac{\left(\omega_{a}^{2}+\omega_{b}^{2}\right)}{2}}\; & A=\frac{\omega_{b}^{2}-\omega_{a}^{2}}{2} \end{array}$$

Finally, we obtain the band-pass filter response for the stop bands (5) and passband (6):

(5) $$|S_{21}{|}^{2}=\frac{1}{1+\delta +\epsilon^{2}cosh\left[\frac{n}{2}cosh^{-1}\left(\frac{\omega^{2}-\omega_{o}^{2}}{A}\right)\right]}$$

(6) $$|S_{21}{|}^{2}=\frac{1}{1+\delta +\epsilon^{2}cos\left[\frac{n}{2}cos^{-1}\left(\frac{\omega^{2}-\omega_{o}^{2}}{A}\right)\right]}$$

where

$$\begin{array}{cc} \; & \omega^{\prime }=\frac{\omega^{2}-\omega_{0}^{2}}{A} \end{array}$$

Note that *n* denotes the number of reactive elements in the transformed filter and is the notation adopted in this study for the filter order.

The numerical values for this bandpass filter are shown in Table 3.

#### 2.2.5. Step 4: Select the Filter Order

A fourth-order topology is selected as a trade-off among matching performance, circuit complexity, and robustness to component tolerances.

To quantify robustness with respect to reflection coefficient $S_{11}$ and bandwidth, we compared fourth- and sixth-order matching networks. A Monte Carlo analysis [38,39] was performed using Gaussian-distributed component variations over 1000 runs.

A tolerance of ±5% is assumed for all off-the-shelf inductors and capacitors of the matching circuit. Tighter tolerances (e.g., ±1%–±2%) are typically associated with reduced availability and higher cost, whereas looser tolerances (e.g., ±10%–±20%) considerably decrease the fraction of candidate networks that meet the specified performance requirements.

**Figure 6:**
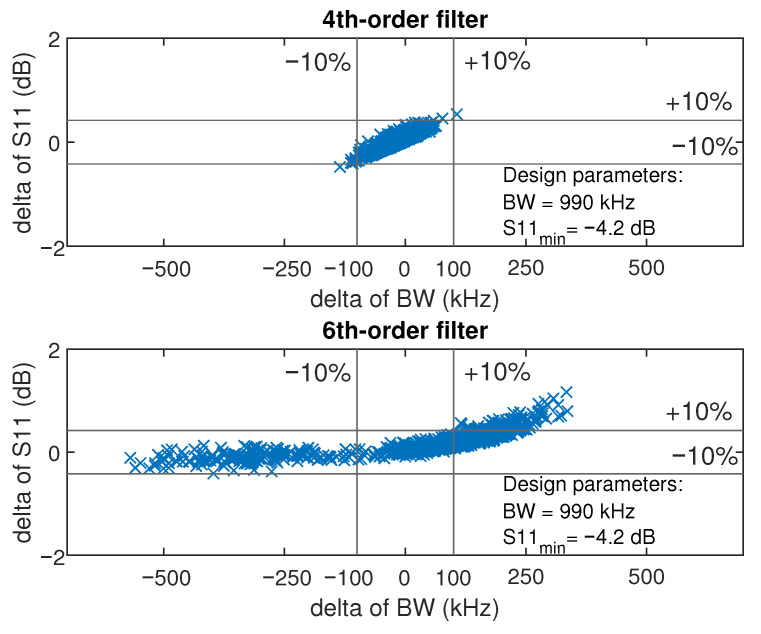
Monte Carlo yield simulation results over 1000 runs for the 4th-order (**top**) and 6th-order (**bottom**) networks.

Figure 6 summarizes the robustness of the matching network against the combined effects of component tolerances. The vertical axis represents the dispersion of the reflection coefficient $S_{11}$ (dB); points near zero indicate simulation runs that approach the nominal (ideal) performance. The horizontal axis represents the dispersion in bandwidth (kHz), where values close to zero likewise correspond to near-nominal behavior. The centered rectangle, bounded by the parallel lines, defines the ±10% acceptance window (pass region) for both metrics. Robustness is quantified by the yield, defined as the fraction of Monte Carlo runs that fall within this pass region.

For an overall component tolerance of 5%, 99% of runs satisfy the ±10% limits for the fourth-order network (top), whereas only 16.5% do so for the sixth-order network (bottom). The reduced yield at higher order is attributed to the larger number of mutual interaction of components tolerances that distorts the filter’s response into a non-Chebyshev conventional format. The asymmetric spread further suggests that increased circuit complexity penalizes bandwidth more strongly than $S_{11}$.

#### 2.2.6. Step 5: Tune the Filter Transfer Function to Satisfy the Bode–Fano Criterion

In theory, a bandpass filter polynomial can be shaped to meet essentially any gain and bandwidth targets. In practice, many of these responses cannot be implemented with a physically realizable passive network. For linear, time-invariant matching networks built only from passive elements, the attainable bandwidth is fundamentally limited by how much power can be transferred to the load, as captured by the Bode–Fano criterion. More generally, Fano [40] derived integral gain-bandwidth bounds that are necessary and sufficient for matching-network realizability with arbitrary loads.

**Figure 7:**
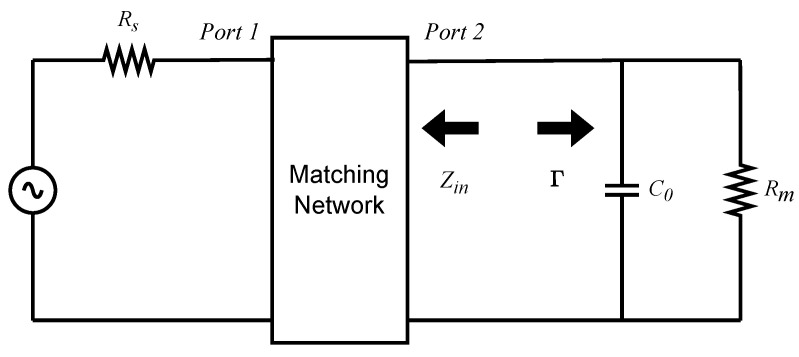
Input impedance and reflection coefficients at the load.

For a load impedance consisting of a resistance *R* in parallel with a capacitance *C*, as in the simplified circuit of Figure 2, the fundamental matching limitation is expressed as

(7) $$\int_{0}^{\infty }ln\frac{1}{\sqrt{1-|S_{21}{|}^{2}}}\;d\omega =\int_{0}^{\infty }ln\frac{1}{\left|\Gamma \right|}\;d\omega \leq \frac{\pi }{RC}$$

where Γ is the reflection coefficient, as shown in Figure 7.

The right-hand-side term is evaluated using the normalized component values as instructed in Step 3. For R=1.0 and C=10.66 (normalized from $R_{m}=4.70\;k\Omega$ and $C_{0}=182.24\;\mathrm{pF}$, respectively), π/RC=0.2947.

The parameter δ in (4) is adjusted to tune $|S_{21}{|}^{2}$ such that the integral in (7) is less than or equal to 0.2947. The value of δ is obtained iteratively following the procedure in [41]. Table 4 lists the resulting δ and $\epsilon^{2}$ values that satisfy the power–bandwidth bound in (7).

#### 2.2.7. Step 6: Compute the Roots of the Reflection Coefficient

We are interested in calculating the poles and zeros of the reflection coefficient obtained by the designed filter polynomial derived previously. The relationship is shown in (8)

(8) $$1-|S_{21}^{2}|=|\Gamma {|}^{2}$$

We first compute the poles and zeros of $|\Gamma {|}^{2}$ for the prototype low-pass transfer function. We then apply the bandpass transformation to obtain the corresponding poles and zeros of the bandpass filter. The roots are computed by applying MATLAB^®^ R2024a (Natick, MA, USA) roots() to the numerator and denominator polynomials of $|\Gamma {|}^{2}$. Only the left-half plane poles are taken, resulting in (9).

(9) $$\Gamma \left(s\right)=\frac{s^{4}+0.42s^{3}+2.21s^{2}+0.48s+1.02}{s^{4}+0.60s^{3}+2.31s^{2}+0.73s^{1}+1.04}$$

#### 2.2.8. Step 7: Synthesize the Matching Network

Network synthesis can be carried out using different canonical forms, as originally established by Foster and Cauer. In this work, we adopt a Cauer Form I realization, which is well suited to the target load impedance and satisfies the imposed electrical specifications and design constraints.

In most impedance-matching formulations, the input impedance ($Z_{\mathrm{in}}$) and reflection coefficient (Γ(s)) are defined at Port 1, i.e., from the source side. Here, instead, we evaluate these quantities at Port 2 by taking the load as the reference, as illustrated in Figure 7. Their general relationship is given in (10).

(10) $$\Gamma \left(s\right)=\frac{R_{m}-Z_{in}}{R_{m}+Z_{in}}$$

From (8), we obtain $Z_{in}$ as shown in (11)

(11) $$Z_{in}\left(s\right)=\frac{1-\Gamma (s)}{1+\Gamma (s)}=\frac{0.09s^{3}+0.05s^{2}+0.12s+0.011}{s^{4}+0.51s^{3}+1.26s^{2}+0.61s+1.03}$$

**Figure 8:**
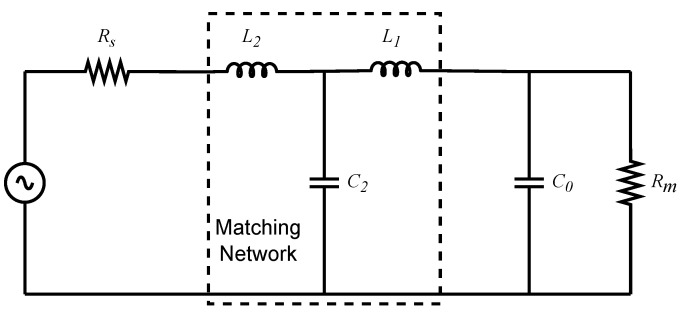
Matched network.

Figure 8 shows a physical system implementation of (11) that is obtained using continued-fraction expansion (Cauer form I [42]). The normalized network values are listed in Table 5, row A.

Finally, component values to be used in further simulations are obtained by frequency and component scaling of the normalized components through (12). The results are listed in Table 5.

(12) $$C_{B}=\frac{C_{A}}{2\pi f_{c}R_{B}}\;L_{B}=\frac{R_{B}L_{A}}{2\pi f_{c}}\;R_{B}=R_{A}R_{m}$$

where $C_{A}$, $L_{A}$, and $R_{A}$ denote the normalized values. The corresponding denormalized component values are $C_{B}$, $L_{B}$, and $R_{B}$.

Once the optimized impedance matching network is designed, the next step is to evaluate its performance in terms of transmitted power and bandwidth through numerical simulations.

### 2.3. Numerical Simulations

In this section, we report simulation results for two analyses: (i) small-signal AC simulations of the power dissipated in the load and (ii) transient simulations used to evaluate the time- and frequency-domain pressure responses to a monopolar pulse excitation. For each analysis, three matching configurations are compared: no matching, single-inductor matching, and broadband matching using the proposed fourth-order Chebyshev network. SPICE simulations of the equivalent-circuit model are performed in Micro-Cap 12 (Spectrum Software, CA, USA) to generate the raw data, and MATLAB is used for post-processing and visualization. In this work, the bandwidth of the Chebyshev Type I filter is defined from the specified ripple limits, rather than from the conventional 3-dB cutoff.

#### 2.3.1. AC Analysis

**Figure 9:**
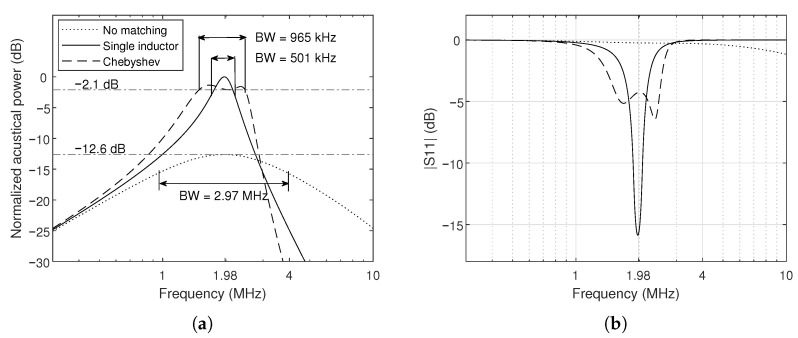
Simulated frequency response for the CMUT without impedance matching, single inductor, and Chebyshev matching, where (**a**) shows the normalized acoustical power at the load, and (**b**) shows the return loss (S11) of the system. The asymmetric shape in the Chebyshev curve is due to non-zero parasitic resistances from the electrical interconnections.

The AC analysis evaluates how well the designed matching network meets the design specifications. Figure 9a shows the normalized frequency response of the acoustic power dissipated at the CMUT load for the three configurations: no matching, single-inductor matching, and Chebyshev matching. The unmatched case exhibits a narrowband response centered at 1.98 MHz, whereas the single-inductor network increases the peak power but further concentrates the response around the resonance. The Chebyshev network broadens the bandwidth while still enhancing the in-band power compared to no matching configuration, consistent with the design goal.

Figure 9b depicts the corresponding |S11| curves for the simplified circuit shown in Figure 2. The Chebyshev network significantly reduces the return loss over a fractional bandwidth close to 50%, demonstrating that the network operates near the Bode–Fano limit for the chosen load parameters.

#### 2.3.2. Pulse Excitation

**Figure 10:**
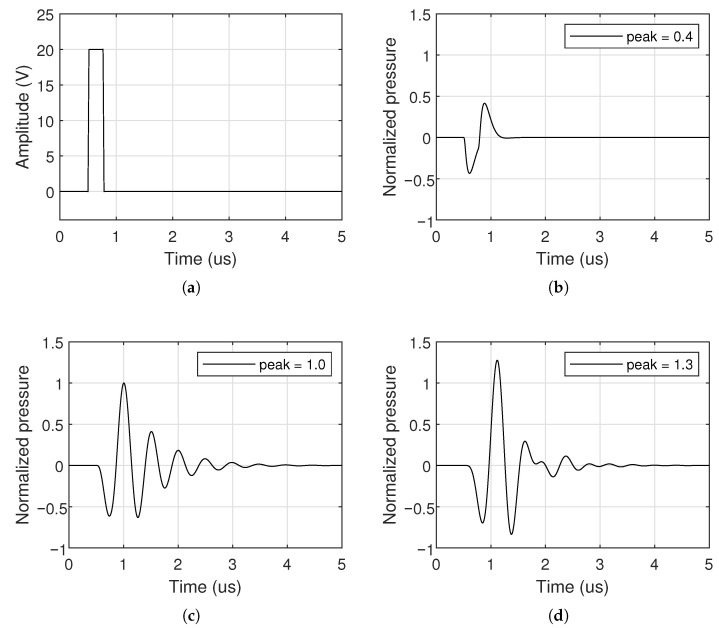
Simulated CMUT transient responses for different impedance matching networks excited by the (**a**) input signal: (**b**) no matching, (**c**) single inductor producing a narrowband response, and (**d**) Chebyshev filter producing a wideband response. The considerably lower ringing from the proposed wideband matching allows shorter input pulses, and improved image resolution.

To assess the effect of impedance matching on typical ultrasound signals, we used a 250 ns unipolar pulse with 20 V_*peak*_ voltage shown in Figure 10a. This pulse length matches the center frequency of the transducers used. The corresponding normalized transmitted pressures for the three matching configurations are plotted in Figure 10b–d.

The unmatched CMUT presents a relatively low pressure level, about 41% of the single inductor pressure. The single-inductor network increases the peak pressure but generates a pronounced narrowband response with longer ringing. In contrast, the Chebyshev network yields both higher peak pressure and reduced ringing, enabling shorter effective pulse durations and potentially better axial resolution in imaging.

**Figure 11:**
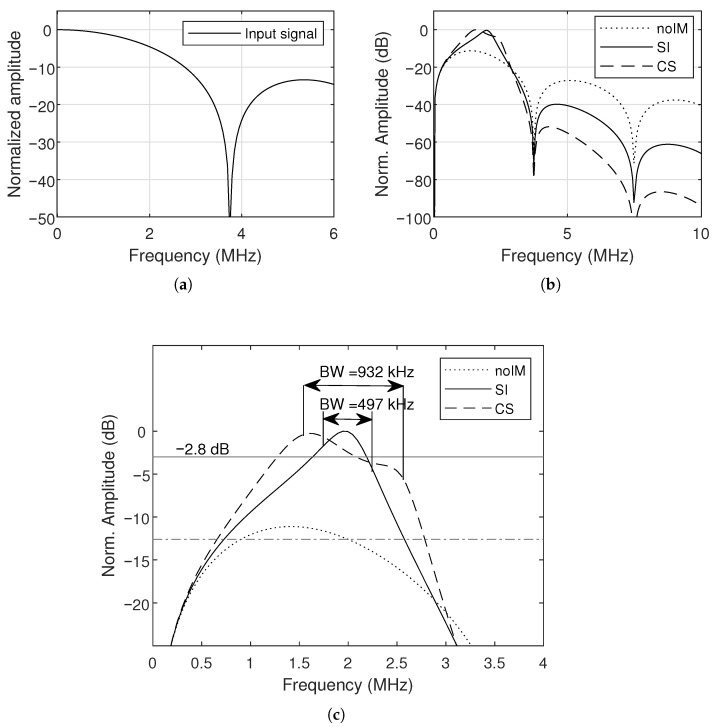
Simulated frequency response for the: (**a**) input pulse, (**b**) CMUT without impedance matching, single inductor, and Chebyshev matching. (**c**) The highlighted region around the resonance frequency.

The spectra of the input and transmitted pulses are shown in Figure 11. The use of unipolar pulses offers a wider bandwidth compared to bipolar pulses, although the peak response is shifted to lower frequencies due to the strong DC component, which cannot be canceled out as in bipolar pulses [43]. This behavior also explains the negative slope response at resonance frequency. The unmatched CMUT has a relatively flat but low-level response around resonance, whereas the single-inductor case exhibits the narrowest spectrum. The Chebyshev network achieves a wider bandwidth while raising the in-band amplitude, confirming its suitability for broadband pulse transmission.

## 3. Results

**Figure 12:**
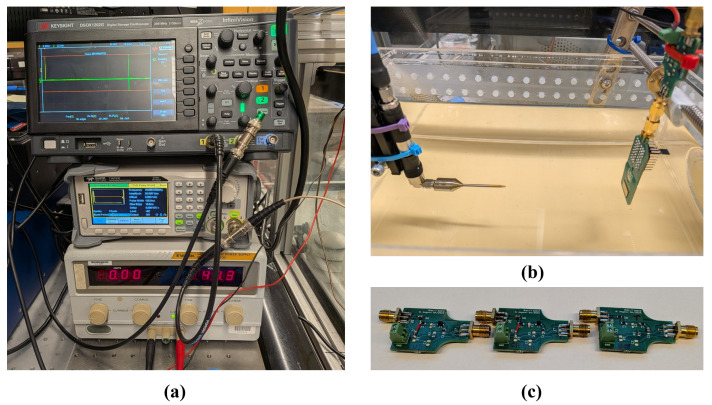
Instrumentation setup for transmission: (**a**) Input source and measurement, (**b**) polyCMUT array immersed and facing the hydrophone, and (**c**) the three matching boards: single inductor (left), Chebyshev (center), and no matching (right).

In this section, we present acoustical measurements from the polyCMUT array (40 V DC bias, immersed in IPA) combined with the different impedance matching networks. The device is evaluated in transmission mode only, where the sound pressure is measured with an hydrophone (HNC-1000, Onda Corp., Sunnyvale, CA, USA) at approximately 10 cm distance from the transducer. We used an arbitrary signal generator (T3AFG30, Teledyne, Thousand Oaks, CA, USA) to generate the pulse, and a digital oscilloscope (DSOX1202G, Keysight Inc., Santa Rosa, CA, USA) to capture the hydrophone signal. The measurement setup is shown in Figure 12.

The components used in the matching circuits of the three boards were selected based on analytically obtained values, as presented in Table 5. These values were derived from the mean impedance and admittance of sixteen measured elements, as detailed in Section 2.2.2. This average-based approach provided the best fit across all channels without the need for individual adjustments, as the standard deviation around 1.98 MHz was sufficiently small to render the associated error negligible (see Table 2). Commercially available components were chosen to be as close as possible to the calculated values, with a tolerance of 5%, since components with lower tolerance are uncommon for inductors in the microhenry range. The remaining components on the three boards constitute the biasing circuit (Bias-Tee), which is identical for all three boards.

A 250 ns unipolar pulse with a 20 V peak amplitude was used for excitation. Although this drive level induces nonlinear behavior, for the voltage range and device parameters considered in this work the BVD model components in Figure 2 remain a valid approximation, with bandwidth errors below 4%.

**Figure 13:**
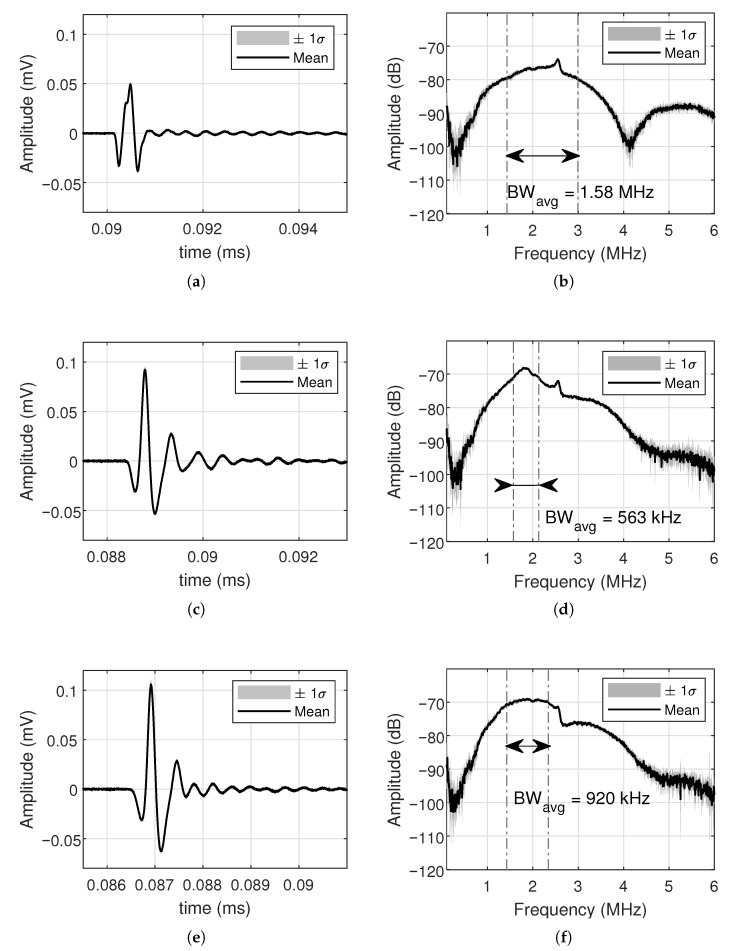
Measured CMUT time and frequency responses for different impedance matching networks excited by the input pulse: (**a**,**b**) No matching, (**c**,**d**) Single inductor, and (**e**,**f**) Chebyshev.

Figure 13 shows the hydrophone output averaged over eight elements, both in time and frequency responses, along with the corresponding ±σ envelope for the unmatched case, single-inductor matching, and the Chebyshev network, respectively.

Table 6 summarizes the gains in acoustic power and bandwidth per each matching network for simulation and experimental averaged results. The data demonstrates that the three matching networks behave as expected: reactive matching structures increase the received acoustic voltage peak at the expense of a reduction in the effective bandwidth of the system.

## 4. Discussion

For the unmatched case, both simulated and measured results are normalized to unity and serve as a reference for the other configurations. The single-inductor matching network yields a moderate increase in the peak acoustic signal, with factors of approximately 1.79 in simulation and 1.86 in measurement. This demonstrates that this narrowband topology concentrates energy around the primary resonance of the transducer. The Chebyshev network provides an even larger enhancement, with gains of 2.25 (simulated) and 2.13 (measured), confirming that the proposed broadband topology improves power transfer to the transducer without excessively degrading the transient behavior of the received signal.

The increase in peak voltage is accompanied by the expected reduction in bandwidth, which is typical for reactive matching networks applied to CMUTs. In simulation, the unmatched system exhibits a bandwidth of 1.74 MHz, which is reduced to 497 kHz with the single-inductor network and to 932 kHz with the Chebyshev network. This shows the intermediate tradeoff the broadband design offers: higher gain than the single-inductor case, while maintaining a significantly broader bandwidth. The hydrophone measurements follow the same trend, with bandwidths of 1.58 MHz (unmatched), 563 kHz (single inductor), and 920 kHz (Chebyshev), indicating that the experimental performance of the Chebyshev network is in good agreement with the analytical predictions.

The discrepancies between simulated and measured results, in both peak voltage and bandwidth, are mainly attributed to deviations between the ideal component values obtained from the design equations and the commercially available components used to implement the matching networks. Small variations in inductance, capacitance, and quality factor shift the resonance frequency and alter the shape of the frequency response, thereby affecting the measured peak gain and bandwidth. In addition, the measured transducers typically exhibit a slightly narrower bandwidth than predicted by the model. This deviation is attributed to non-ideal mechanical boundary conditions, which lead to a mild attenuation of the higher frequency components [29]. It should also be noted that the hydrophone does not provide a perfectly flat frequency response across the frequency range of interest. Above approximately 2.5 MHz, the sensor shows a modest increase in sensitivity caused by these effects, which appears as a small bump in Figure 13b,d,f.

Despite these discrepancies, the results validate the proposed design strategy: the Chebyshev matching network simultaneously provides higher peak gain and wider bandwidth than the single-inductor network, making it more suitable for applications requiring broadband acoustic signals with high transmission efficiency. On the other hand, the fact that the measured bandwidth is slightly smaller than the simulated one, particularly for the unmatched case, indicates that a complete characterization of the system must include not only the electrical model of the transducer and matching network but also the detailed calibration of the hydrophone frequency response and the tolerance of the passive components employed.

Although this work focuses on transmit operation, the proposed passive matching network may also be advantageous in receive mode, since passive matching networks are generally reciprocal. The ability to sustain wider bandwidth than traditional narrowband passive matching is particularly relevant to medical imaging, where increased bandwidth enables shorter pulses and, consequently, improved axial resolution. However, replacing conventional receiver front-end electronics with a purely passive network requires additional analysis. In particular, transimpedance amplifier (TIA) front ends can effectively compensate the CMUT’s shunt capacitance $C_{0}$, albeit at the expense of increased power consumption and added noise.

While the proposed approach is applicable to CMUTs with different geometries, materials, and target applications, the need to match a complex load to a finite source impedance constrains the feasible filter type and order. In particular, higher-order networks require a larger number of components and tighter tolerance control, which increases design complexity, cost, and sensitivity to implementation errors.

The practical implementation of the proposed matching approach is also constrained by front-end electronics integration limits, including the available channel count and the transducer footprint, especially when higher-order networks are required. A potential workaround would be the use of dedicated printed circuit boards with embedded inductors and capacitors, which could alleviate the space limitations and improve integration, but such an approach lies beyond the scope of the present study. Nevertheless, the proposed technique is well suited to systems with a limited number of channels, since it provides a controllable trade-off between bandwidth and transmitted power under specific operating conditions.

## 5. Conclusions

This work presents a complete and systematic workflow for the design of passive broadband impedance-matching networks for immersed polyCMUT arrays. Three configurations were designed and experimentally evaluated: unmatched operation, single-inductor matching, and a Chebyshev broadband network guided by the Bode–Fano limits. Hydrophone measurements demonstrate that the Chebyshev network provides the best overall performance, achieving a 2.13-fold increase in peak acoustic signal relative to the unmatched case while maintaining a broad bandwidth of 920 kHz at a center frequency of 1.98 MHz. In comparison, the single-inductor network yields a peak gain of 1.86 with a reduced bandwidth of 563 kHz, whereas the unmatched configuration offers the widest bandwidth of 1.58 MHz but at substantially lower peak output.

These results confirm the expected power–bandwidth trade-off of reactive matching, but also show that a properly designed broadband network can boost transmit output without becoming overly narrowband. The close agreement between simulations and experiments obtained in this study indicates that the model and design flow are reliable, with remaining gaps mainly explained by real component tolerances and losses.

The main contribution is a practical, repeatable matching methodology for polyCMUT arrays in immersion, enabling designers to choose bandwidth vs. power intentionally. This supports higher-output CMUT systems for applications where drive voltage, size, and power consumption are constrained.

## Figures and Tables

**Table 1:** Layer definitions and thickness.

Layer	Thickness (μm)
Bottom electrode	0.100
Silicon Dioxide	0.005
Cavity (LOR 1A)	0.176
First polymer (SU-8)	0.450
Top electrode	0.100
Second polymer (SU-8)	4.20
Passivation (Parylene-C)	4.10

**Table 2:** Measured key characteristics of the polyCMUT array ± single standard deviation.

Parameter	Measurement
Resonant frequency $f_{r}$	1.98 ± 0.013 MHz
Resistance $R_{par}$	13.79 ± 1.23 Ω
Capacitance $C_{0}$	182.24 ± 2.71 pF
Conductance $G_{p}$ at $f_{r}$	287.43 ± 10.4 μS
Susceptance $B_{p}$ at $f_{r}$	2.33 ± 0.028 mS

**Table 3:** Normalized bandpass filter parameters.

A	$\omega_{a}$	$\omega_{b}$	$\omega_{o}$
0.5	0.75	1.25	1.0308

**Table 4:** Tuning parameters.

δ	$\epsilon^{2}$
0.6150	0.347

**Table 5:** Component values of the resulting matching network. Row A for the normalized value, row B for the final value.

	$R_{s}$	$L_{2}$	$C_{2}$	$L_{1}$	$C_{o}$	$R_{m}$
A	0.0106	0.0208	44.7919	0.0978	10.6558	1
B	50 Ω	7.87 μH	766 pF	36.94 μH	182.24 pF	4700 Ω

**Table 6:** Comparison between simulated and experimental results.

	Transient		Frequency	
	Simulation	Measurement	Simulation	Measurement
	V_*peak*_ Increase	V_*peak*_ Increase	Bandwidth	Bandwidth
No matching	1	1	1.74 MHz	1.58 ± 0.05 MHz
Single inductor	1.79	1.86	497 kHz	563 ± 45.8 kHz
Chebyshev	2.25	2.13	932 kHz	920 ± 39.5 kHz

## Data Availability

The data presented in this article are available on request from the authors.
